# Supplementary figures and images for: Task-related activity in human visual cortex
Source: PLoS Biol. 2020 Nov 6;18(11):e3000921. doi: 10.1371/journal.pbio.3000921 (PMC7673548; doi:10.1371/journal.pbio.3000921)

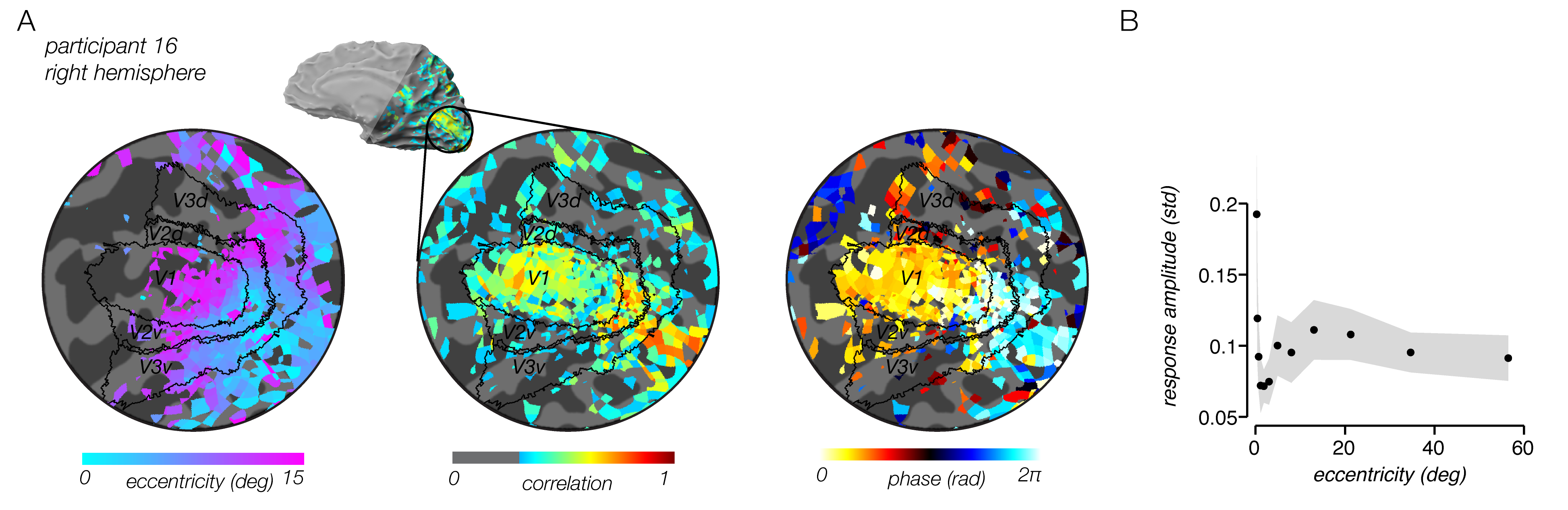

Supplement: S1 Fig — (A) Medial view (inset) and a flattened map of right hemisphere visual cortex (bottom panels) of participant P16. Left: visual eccentricity. Hue indicates preferred eccentricity for each voxel. Retinotopic borders of V1–V3 were defined by an anatomical template extending to 80° eccentricity, well beyond the spatial extent of the screen. Map threshold, r > 0.3. Shaded region on lateral and medial views indicates cortex not included in the imaged/field of view. Center: response correlation for control experiment, showing a widespread fMRI response linked to task timing. Map threshold, r > 0.3. Hue indicates correlation with best-fitting cosine at the task frequency. Right: response phase. Same threshold as middle panel, with hue indicating phase of best-fitting cosine for each voxel. Phase values indicate the response latency for each voxel. (B) EVC task-related response amplitude as function of eccentricity. Shaded regions, ±SEM across participants. Amplitude varies with eccentricity in a similar manner to the main experiment; compare with Fig 4A. EVC, early visual cortex; fMRI, functional MRI. (TIF) [file pbio.3000921.s001.tif]

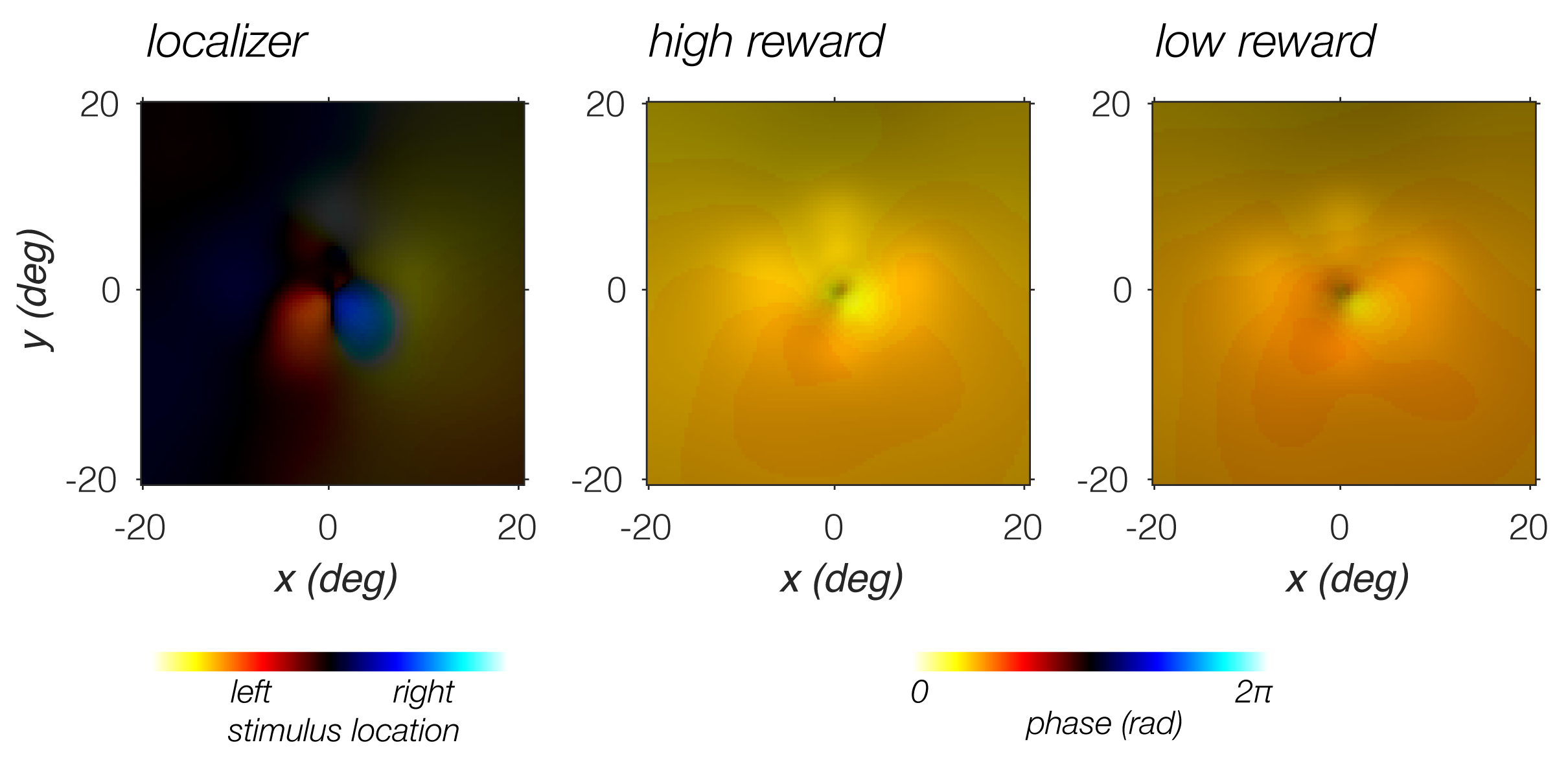

Supplement: S2 Fig — Phase and coherence of visual cortex responses retinotopically projected back to the visual field, averaged across n = 14 participants. Opacity of each pixel reflects the coherence of the time series obtained by averaging across voxels, weighted by their retinotopic response to that pixel. Hue reflects the phase of the resulting time series. Left, average of localizer runs. Center, average of high-reward runs. Right, average of low-reward runs. Voxels are from combined right- and left-hemisphere EVC ROIs. Localizer runs evoked localized activity limited to voxels with pRFs that overlap the stimulus. In contrast, task runs evoked widespread activity that did not correspond retinotopically to the stimulus. The inverted response is not visible in high- and low-reward runs because of the small size of foveal pRFs. Foveal voxels have small pRF sizes, overlapping with more peripheral pRFs at different phases. This results in low coherence at the fovea, i.e., a small opaque area at the center. EVC, early visual cortex; pRF, population receptive field; ROI, region of interest. (TIF) [file pbio.3000921.s002.tif]

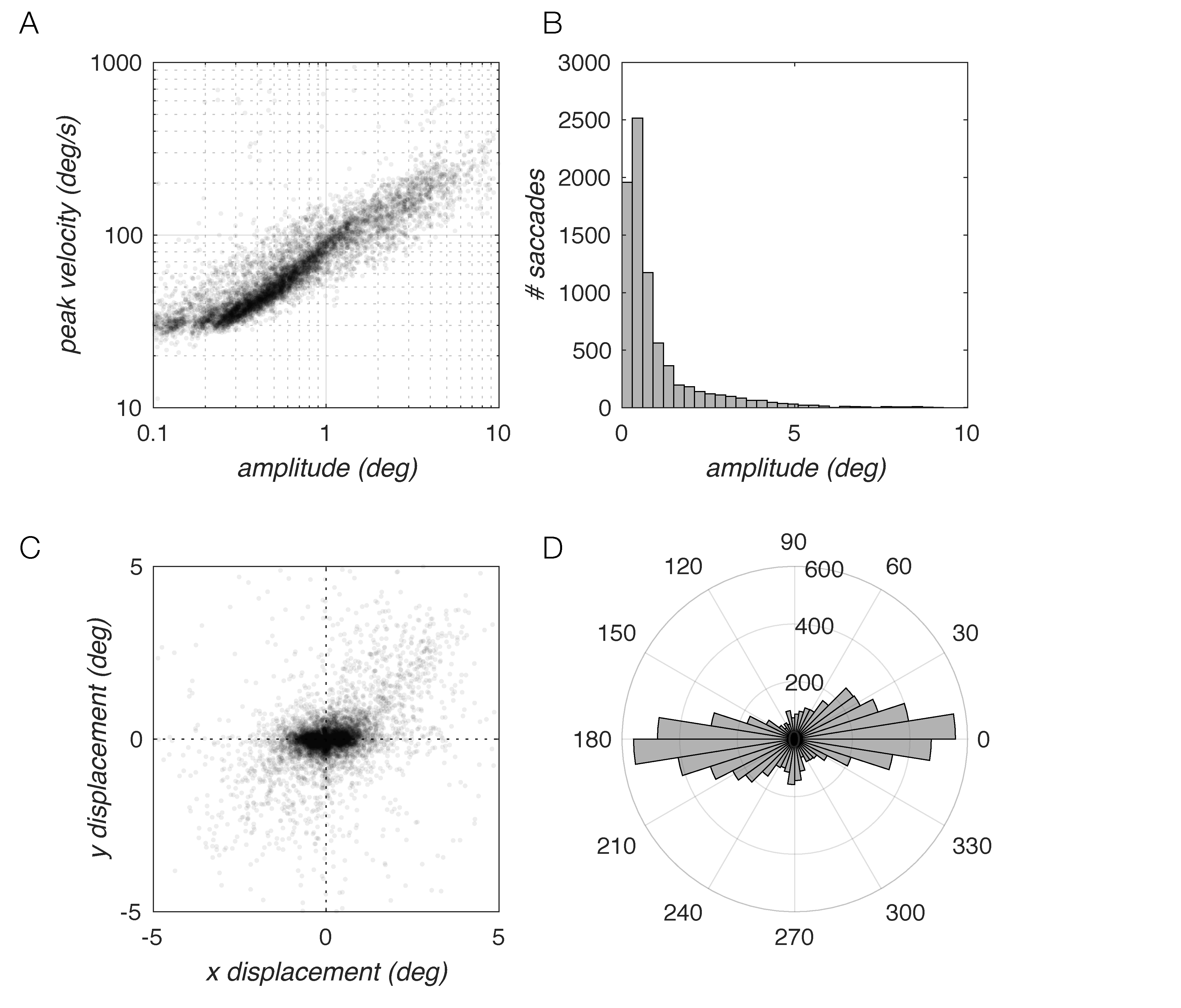

Supplement: S3 Fig — Saccades occurring within the first 1,000 ms of each trial were analyzed. (A) Main sequence, pooling saccades across participants, demonstrates a linear relationship in log-log axes between peak velocity and saccade amplitude, as expected by the biomechanics of the oculomotor plant. (B) Amplitude distribution of saccades. Most saccades were small (<1 deg) and are hence considered microsaccades. Median saccade amplitude, 0.52 deg. (C) Spatial distribution of saccades. Each dot represents the displacement of a saccade relative to the origin (0,0). (D) Direction distribution of saccades. Saccades are generally horizontal and were not directed toward the target, nor were they of sufficient amplitude to reach the target. (TIF) [file pbio.3000921.s003.tif]

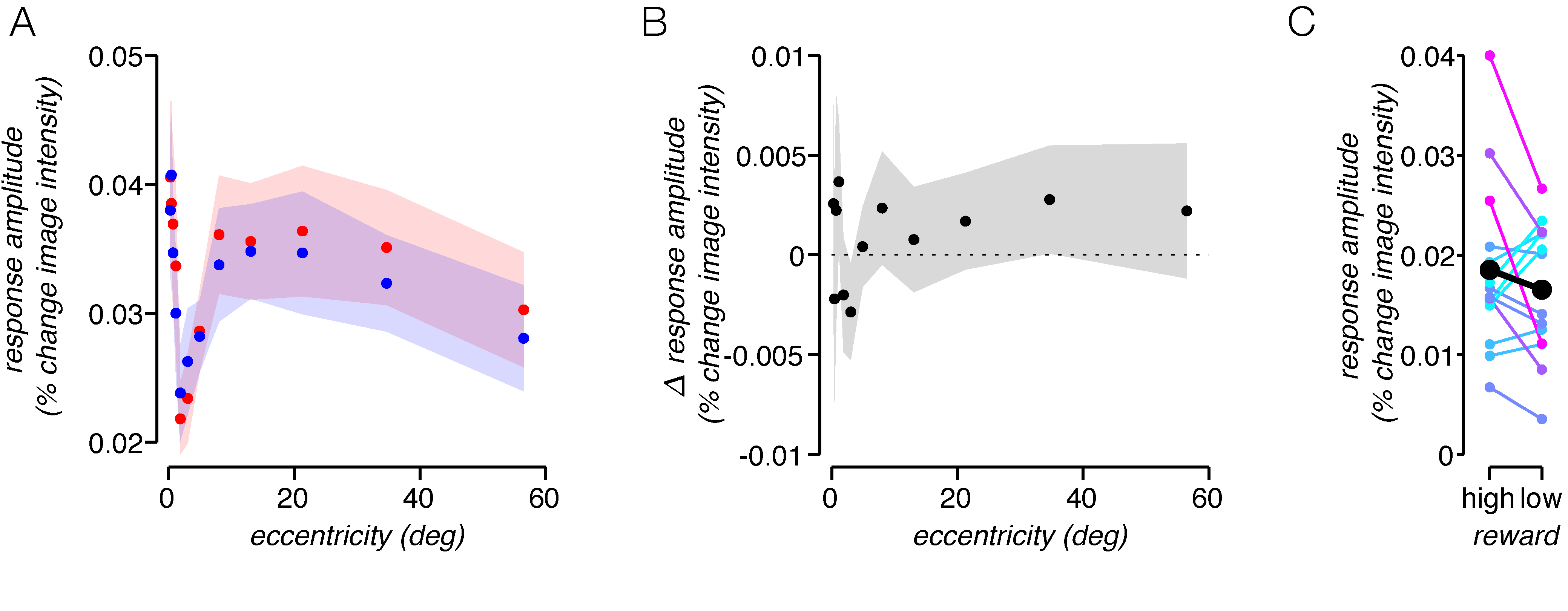

Supplement: S4 Fig — For this supplementary analysis, time series were not z-scored. In all other respects, analysis and figure are identical to Fig 4. High reward had significantly higher amplitude (p = 0.004) and lower time-point variability (p = 0.0012), temporal variability (p = 0.0218), and amplitude variability (p = 0.0423). fMRI, functional MRI. (TIF) [file pbio.3000921.s004.tif]

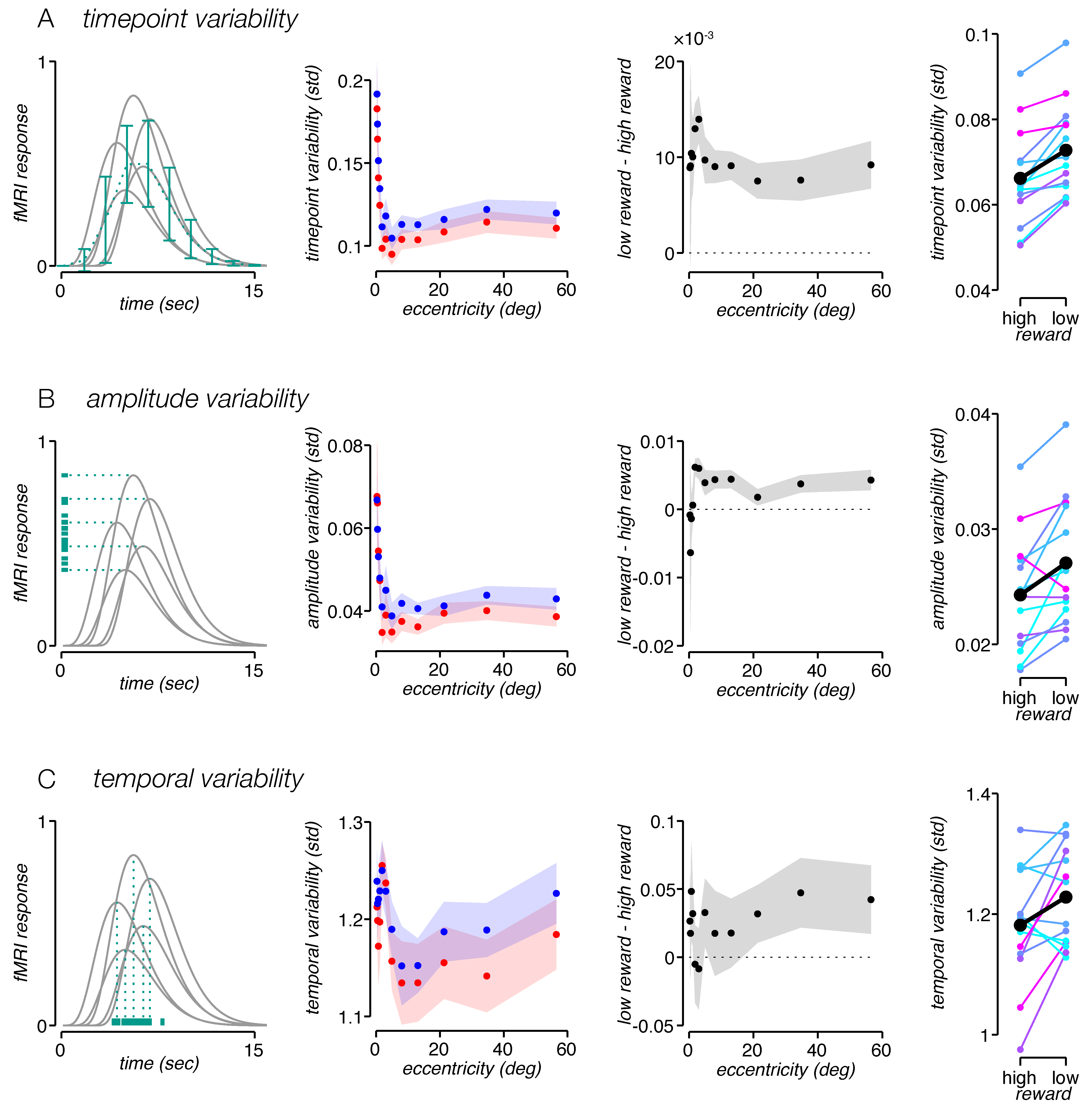

Supplement: S5 Fig — For this analysis, time series were not z-scored. In all other respects, analysis and figure are identical to Fig 5. High reward had significantly lower time-point variability (p = 0.0012), temporal variability (p = 0.0218), and amplitude variability (p = 0.0423) than low reward. Time-point variability was significantly greater for low reward in each of the eccentricity bins (p < 0.01 for all). fMRI, functional MRI. (TIF) [file pbio.3000921.s005.tif]

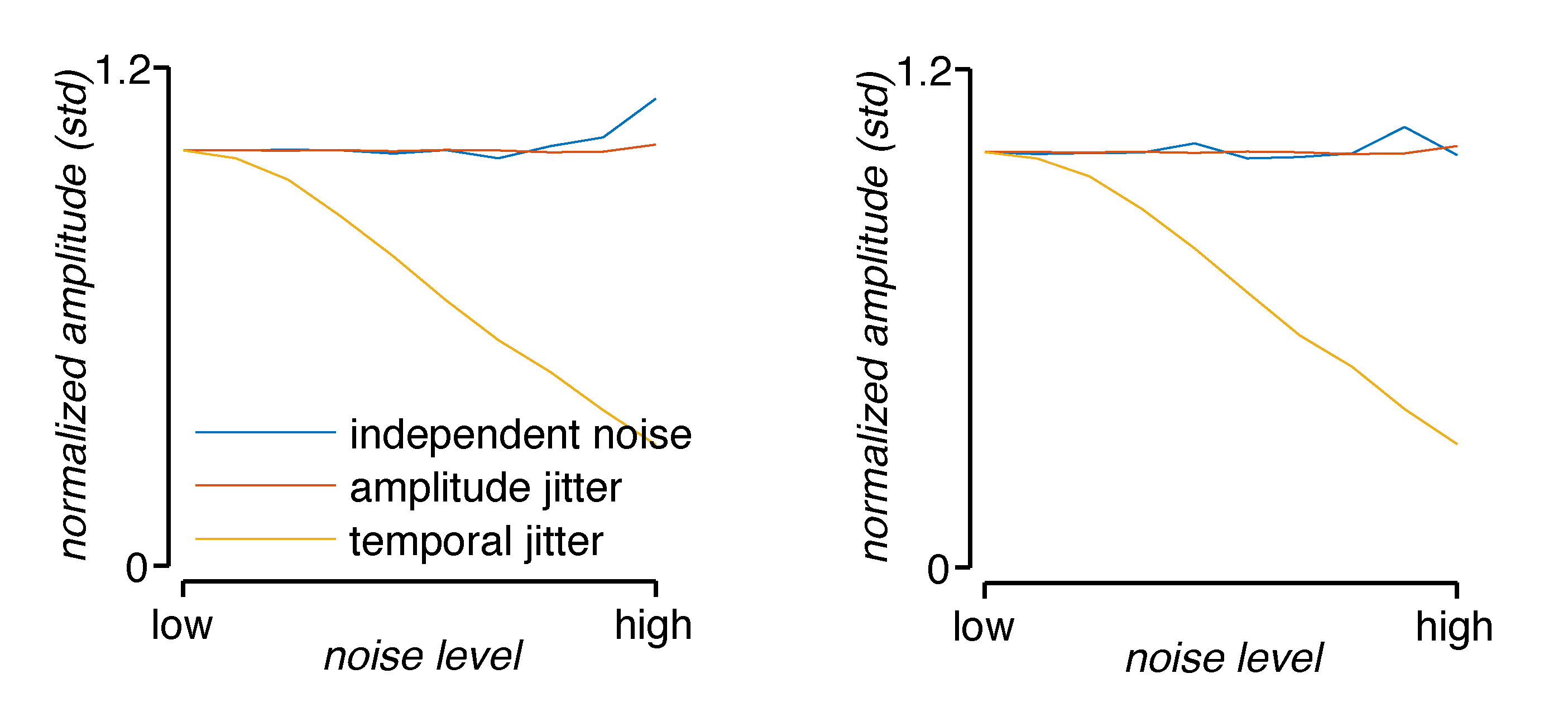

Supplement: S6 Fig — Simulated response amplitude as function of noise level for first (left panel) and second (right panel) simulations. Amplitude was computed as the standard deviation of the time series presented in Fig 6 and normalized relative to amplitude at noise level 1 (i.e., no noise). Amount of independent noise and amplitude jitter had no systematic impact on response amplitude, whereas amplitude drops monotonically with increasing temporal jitter. (TIF) [file pbio.3000921.s006.tif]
