# Supplementary material for: Task-related activity in human visual cortex
Source: PLoS Biol. 2020 Nov 6;18(11):e3000921. doi: 10.1371/journal.pbio.3000921 (PMC7673548; doi:10.1371/journal.pbio.3000921)
Supplement: Attachment — Submitted filename: PLOS_reviewers.docx [file pbio.3000921.s007.docx]

We thank the reviewers and the academic editor for their thoughtful comments. The manuscript has been substantially revised in accordance with each point raised in their reviews. Below, reviewers comments are indicated by **bold typeface.** For each point, we summarize our response, and indicate locations of changes to the manuscript.

**Your revisions should address the specific points made by each reviewer. Having discussed these comments with the Academic Editor, four things are more salient to us and should be addressed thoroughly:**

**1) The claim of no stimulus driven effect by an ipsilateral stimulus. We know that feature-selective modulations can spread to the ipsilateral regions, so this assumption needs careful justification.**

We completely agree. We have taken a number of steps to support the claim of no stimulus driven effect in ipsilateral cortex, including new data and additional analyses. Please see our detailed response below, under Reviewer 1, point 1.

**2) The issues about variability in each condition and the dependent measure of the BOLD response also need careful consideration.**

We have repeated all of the analyses without normalizing the variability of the BOLD time series data (i.e., without z-scoring) and found that the results remain significant. We have included a discussion of this point below, under Reviewer 1, points 2 & 3.

**3) Reviewer 2 expressed important qualifications that need to be included about valance/arousal**

We have added a section to the Discussion (*Discussion: Manipulating arousal with reward*) addressing this issue. Please see our response under Reviewer 2, point 1.

**4) Reviewer 2 also wondered about stimulus driven responses - this seems fairly important to cover more thoroughly.**

We have added a section to the Discussion (*Discussion: Effect of arousal on stimulus-evoked activity*) addressing this issue. Please see our response under Reviewer 2, point 2.

------------------------------

**Reviewer #1, Kristina Visscher: Summary:**

**Roth et al., investigate the relationship between arousal and task-related neural activity in visual cortex. The authors measure fMRI signals in V1 during an orientation discrimination task, while simultaneously recording pupil size and heart rate as measures of arousal. They tested the hypothesis that task-related evidence tracks arousal.**

**First they show that pupil size and heart rate change during trials of the task.  They show also that global mean regression removes the signal associated with pulse.  A selective averaging type approach was used to measure the level of fMRI activity in response to trials, in units of standard deviation of BOLD signal across the run duration.  Trial by trial variability in timepoint, amplitude and time to peak were smaller in the high than the low reward case.**

**Another interesting result was that the amplitude (and, to a lesser degree, variability) of MRI response was very small at brain voxels corresponding to ~2-4 degrees eccentricity, but was larger at smaller and at larger eccentricities.  This 'dip' is on its face unexpected, and the authors give a number of possible interpretations of these data in the discussion section. The authors also perform a simulation to show how trial to trial variability in various aspects of neural activity could lead to changes in the standard deviation of a signal.**

**The major inferences from these results are that:**

**1) Because the response follows dynamics similar to that of pupil size and heart rate, task-related activity is related to arousal**

**a. I think this isn't a very strong argument; granted, the authors do hedge, and use the term "suggest."  However, it seems reasonable from first principles (and because the pupil size and heart rate change with reward condition) that the high reward and low reward conditions could differ in arousal.  That's the only argument I think is needed to make later arguments about arousal being a likely factor contributing to the observed effects.**

**2) Higher reward increased response amplitude (measured in Fig 3) and decreased trial to trial variability (Figure 5, right side), suggesting that arousal (reward task) decreases trial-to-trial variability in responses.**

**3) There is a very interesting and hard to interpret result shown in Figure 4, where response amplitude depends in an interesting way on eccentricity.**

**Major:**

*** The authors assert that the changes in neural activity observed during the orientation discrimination task was due to arousal.  However, it seems that there may be reason to believe that the changes are likely due to other top-down influences outside of arousal. The logic of the paper rests on this idea, e.g., from line 333 in discussion "This activity is not evoked by the stimulus, since there was never a stimulus contralateral to the hemisphere we analyzed."**

**There needs to be stronger evidence that activity ipsilateral to a stimulus is due to arousal and not, e.g., activity driven by the strong functional coupling between mirror symmetric portions of cortex, including V1.  If there was activity in response to the stimulus in the contralateral side, it seems reasonable to suppose that there would be activity in response to that activity in the highly functionally correlated opposite side.  The authors should provide description of why the argument that activity in ipsilateral visual cortex could not come from ipsilateral stimulation.  [of course, ipsilateral activity would be far less than contralateral activity. But for the connectivity reasons given above, and due to small effects in other datasets, it seems reasonable to suppose that some activity in ipsilateral cortex could be predicted.]**

We agree that it is important to show that we are not studying stimulus-evoked activity. We designed the study to minimize the impact of the visual stimulus, and although the stimulus was small, brief, and peripheral, and only appeared in the right visual field (RVF), it is conceivable that the stimulus itself contributed to the activity that we measured in in the ipsilateral (right) hemisphere in the main experiment. While we think this possibility is remote, as Reviewer 1 notes, numerous studies have reported a pattern of homotopic functional connectivity in visual cortex, in which mirror symmetric portions of visual cortex exhibit time series correlations. Such connectivity could, in principal, result in ipsilateral responses to the stimulus as Reviewer 1 describes.

We have taken the following steps to demonstrate that the task-related activity is not related to the stimulus.

First, we have included data in the revision from an experiment in which subjects performed a periodic button press task that had the same timing as the task in the main experiment, but did not involve a peripheral visual stimulus (*Methods: Stimulus-free experiment*). This experiment is very closely related to the optical imaging experiments in monkey by Sirotin and Das (*Nature*, 2009). We observed the same widespread task-related response extending from the fovea to the most peripheral representation (Fig S1). This observation suggested that the task-related response in the main experiment was not driven by the ipsilateral visual stimulus.

***Fig S1. Task-related activity in visual cortex in the absence of visual stimulus.*** *(A) Medial view (inset), and a flattened map of right hemisphere visual cortex (bottom panels) of subject S16. Left: visual eccentricity. Hue indicates preferred eccentricity for each voxel. Retinotopic borders of V1-V3 were defined by an anatomical template extending to 80° eccentricity, well beyond the spatial extent of the screen. Map threshold, r>0.3. Shaded region on lateral and medial views indicates cortex not included in the imaged /field of view. Center: response correlation for control experiment, showing a widespread fMRI response linked to task timing. Map threshold, r>0.3. Hue indicates correlation with best-fitting cosine at the task frequency. Right: response phase. Same threshold as middle panel, with hue indicating phase of best fitting cosine for each voxel. Phase values indicate the response latency for each voxel.(B) EVC task-related response amplitude as function of eccentricity. Shaded regions, ± SEM across subjects. Amplitude varies with eccentricity in a similar manner to the main experiment; compare with Fig 4A.*

Second, we analyzed data from a localizer scan that was run on every subject and in every scanning session of the main experiment (*Methods: Stimulus localizer*). In this localizer, we measured fMRI responses to the same RVF stimulus as in the main experiment, and, on separate trials, responses to the stimulus presented in the mirror symmetric location in the LVF. The stimuli in the localizer scan were presented for a longer duration (9 s instead of 200 ms) in order to evoke stronger, clearer visual responses. We then repeated all of the analyses in the main experiment, excluding voxels that responded to either of the two stimuli in the localizer experiment. We found that all of the main results remained significant after excluding these voxels. This analysis demonstrates that the impact of reward on task-related fMRI activity occurs at cortical locations that are not activated by the appearance of the visual stimulus in the ipsilateral hemifield, or its mirror symmetric location in the contralateral hemifield.

Third, we have used estimates of the receptive field location for each voxel to create visual field coverage plots from the population responses in early visual cortex. We created these plots for the data in the visual localizer experiment, which included RVF and LVF stimuli (and no task), and for the main experiment, which included only a brief RVF stimulus (and a task). These plots show that task-related activity is far more spatially extensive than visually-evoked activity (Fig S2).

*Fig S2. Task-related activity is distinct from visual activity. Phase and coherence of visual cortex responses retinotopically projected back to the visual field, averaged across n=15 subjects. Opacity of each pixel reflects the coherence of the timeseries obtained by averaging across voxels, weighted by their retinotopic response to that pixel. Hue reflects the phase of the resulting timeseries. Left, average of high reward runs. Center, average of low reward runs. Right, average of localizer runs. Voxels are from combined right and left hemisphere EVC ROIs. Localizer runs evoked localized activity limited to voxels with pRFs that overlap the stimulus. In contrast, task runs evoked widespread activity that did not correspond retinotopically to the stimulus.*

*** Response amplitude is measured in std.  Why is this choice of units used?  It seems to be the STD across the whole run.  Thus, on a run with higher variability due to task (like the high reward condition), we would expect the total STD to be larger.  So the excursion due to any given trial would be artificially smaller in that case.  I think it warrants giving a clear justification of why STD was used instead of % BOLD change, or another measure, especially because (next bullet point), it appears that this choice could result in false positives. In particular, why do MRI responses not start at time 0 with value = 0 (fig 3, they all seem to be significantly different from 0).**

We feel that z-scoring the time series within a run is justified (see below). However, we have reanalyzed all of our data and found that the results remain significant when z-scoring is not performed.

In fMRI scans of phantoms, the variance in the time series can change from one run to the next, which indicates that differences in run-to-run variance can, in part, be due to machine noise. Z-scoring the time series removes the impact of this noise. But, as the reviewer points out, z-scoring could in principle also introduce spurious differences between conditions. To address this concern, we have added to the supplement versions of all of the figures created from data that have not been z-scored (Fig S4-S5).

The reviewer wondered why the responses don’t start at 0. This is a result of subtracting the mean. After subtracting the mean, the average of the timeseries (and of the average trial) is 0. This is typical of periodic experimental designs, such as in retinotopy. Responses can (and do) start below zero when their peaks are above zero.

*** Because there is a smaller amplitude of signal in the low reward case, the STD units in that case would correspond to smaller numbers in units of % change in BOLD (for example).  Thus, the same change in BOLD would correspond to a larger STD value for the low reward case than the high reward case.  Thus, the same variability in BOLD would be a larger in low reward than high reward case.  Can you show that this simplistic explanation does not explain the data from Figure 5?  One way to do this would be to re-calculate the values in terms of % change from baseline (where baseline is defined as activity at time 0, not as mean across all signals -- standard GLM analysis would work).  There may be other logical ways to get around this issue, but the bigger picture is: how do we convince ourselves that the effect in Fig 5 is not due to the choice of units.**

We agree with the reviewer’s point. We have followed the reviewers suggestion and calculated the values in terms of % change. In the reanalysis of our data, the only steps taken in processing the data are subtracting the mean of the time series, dividing by the mean, and multiplying by 100, resulting in units of % change in image intensity.

*** One of the major interpretations from the data seems to be that trial to trial variability decreased with reward.  And all the figures show response amplitude differences that depend on eccentricity (a lot).  And yet, there are no plots showing how timepoint variability changes with eccentricity.  This seems like it might lend support to the arguments, or at least be informative about the origins of this effect.  The authors should add that figure, or explain why it is not included.**

Thank you for this suggestion. We have added panels showing variability as a function of eccentricity (Fig 5, second column).

**Minor:**

*** fits to the models needed to measure amplitude**

We have added plots of the simulated response amplitude as a function of noise level (Fig S6). We believe these figures elucidate the finding that increasing the amount of temporal jitter decreases response amplitude, while changing the amount of amplitude jitter, or the amount of independent noise, has no impact on response amplitude.

**Fig S6. *Simulated response amplitude as function of noise level for 1^st^ (left panel) and 2^nd^ (right panel) simulations****. Amplitude was computed as the standard deviation of the time series presented in Fig 8, and normalized relative to amplitude at noise level 1 (i.e. no noise). Amount of independent noise and amplitude jitter had no systematic impact on response amplitude, whereas amplitude drops monotonically with increasing temporal jitter.*

*** I assume trials were presented one per 15 seconds (based on doing some division based on lines 579 to 580).  Did I miss this point?  Was a GLM used for data analysis?**

Yes, 1 trial per 15 seconds. The text now reads: “Each trial lasted for 15 s. Each trial began with the appearance of a small oriented grating for 200 ms. Subjects determined whether the grating was tilted clockwise or counterclockwise relative to vertical and responded with a button press. The interstimulus interval was 14.8 s.”

We did not use a GLM for data analysis, since we did not know beforehand the temporal profile of the task-related response and we wanted to follow an analysis strategy that would accommodate a range of response profiles.

*** How many trials per condition were included?  (run is 16 trials, there were 20 sessions.  But how many runs per session?)**

The number of runs per session varied a bit across subjects. Most subjects completed 12 runs per session (6 runs for high reward and 6 runs for low reward), plus 1-2 localizer runs. The minimum number of runs completed for the task experiment was 10. The maximum was 16. We have clarified this in the text (*Methods: fMRI experiment*).

*** Should line 578-579 read "total of 20 sessions in each of 15 subjects"?**

The text is correct. There were a total of 15 subjects in this study, some of whom were scanned in multiple sessions.

*** In line 322, "the difference between the two exhibits a temporal profile similar to each of the two reward conditions" -- it's unclear (at least to this reader) what the temporal profile being described is.  It implies that Figure 7B is similar to another figure, but it's unclear which figure?  (is it 3B?  I don't see the similarity)**

The difference in timepoint variability between high reward and low reward (Fig 7B) is similar to high reward and low reward timepoint variability time series (Fig 7A). The text now reads: “*and the difference between the two exhibits a temporal profile similar to temporal variability for each of the two reward conditions (Fig 7A).”*

*** In line 352, it is noted that the modulation of task related responses with reward resembled modulation of pupil size.  Please indicate which data are used for that conclusion.  If the argument is that pupil size effects are larger in high than low arousal conditions, and fmri effects are larger in high than low arousal conditions, this isn't compelling evidence that "task-related activity is related to arousal."**

The similarity we note refers to the observation that both the task-related response (Fig 4) and pupil size (Fig 2A) increase in amplitude when reward is greater. We have revised the text to make this clearer.

We realize that the link between changes in the task-related response and pupil size (or arousal) isn’t necessarily causal. However, an increase in reward consistently impacts – and in a similar way – both pupil size and task-related activity, suggesting a link between the two. We have added a qualifying statement to the Discussion section clarifying this point (*Discussion,* *first paragraph)*.

*** Around line 474, the text refers to the shape of the effect exemplified in Figure 4a as "an inverted task-related response in and around the foveal representation" -- I find this a confusing characterization, since the very small eccentricities (around 0) have strongest responses, while the areas outside (there isn't an axis label, but it looks like around 4 degrees -- which is technically outside the fovea) have the weakest responses.  Why not call it "a dip in response around eccentricity of 4 degrees" or something like that.  The characterization as it stands suggests that there is no response at fovea, when the largest response is at fovea.**

The inversion of the task-related response at the fovea refers to the difference in phase (response timing) between responses at the fovea versus the periphery. A consequence of this inversion is the profile of response amplitude across cortex, which follows a U shape, rather than a ramp, increasing at higher eccentricities. We point out the difference between fovea and periphery with regard to the brain map of response phase in Fig 1B, and with regard to response within 3 eccentricity bins in Fig 3. We discuss this inversion again in text associated with Fig 4A, which shows mean amplitude as a function of eccentricity. There is a drop in amplitude at around 4 degrees. This reflects a transition from one response phase at the fovea to an opposite phase in the periphery. At the boundary of the fovea, where the two phases meet, there is destructive interference of the response waveforms, causing a diminished response amplitude. This dip in amplitude is a consequence of the inversion in phase.

*** Line 484 refers to the "oft-characterized time course of microsaccade rate modulation during a range of cognitive tasks" -- citation please.**

We added the relevant citations for the rate modulation. We have also added a figure showing the spatial distribution of microsaccades in the 1000 ms following stimulus onset (Fig S3).

*** The section about "heterogeneous task-related response phase" gives good arguments against each of the described explanations, except the last one.  It would be worth more detail on the last item if it is the most likely possible explanation.**

We also thought that the final explanation made most sense. However, after the analyzing the data from the stimulus-free control experiment for this revision, we no longer believe that this final explanation is the most likely. In the control experiment there is no peripheral stimulus, yet we still observed a clear inversion of the response around the fovea (Fig S1, see above, in response to Reviewer 1 point 1). We added the following in the text: *“However, we observed a similar inversion of the response at the fovea during a task with no peripheral stimulus, where subjects had no incentive to shift attention to the periphery (Fig S1). This suggests that the peripheral stimulus, and attention to it, are not the cause of the inversion of the foveal task-related response. Nevertheless, it is possible that even without a peripheral stimulus the periodic task at fixation evoked periodic foveal attention that differed in timing from the task-related response, causing the inverted response at the fovea.”*

*** In line 60, the authors state that their hypothesis is that "task-related evidence tracks arousal." The use of the word "evidence" seems an odd word choice.  It may be worth changing this statement to task-related responses tracks arousal**

We corrected the sentence to read: *“We tested the hypothesis that task-related activity tracks arousal.”* Thank you for catching that error.

*** In line 79 , since this appears to be the first use of the abbreviation "CW" and "CCW", they should be written out as clockwise (CW) and counter clockwise (CCW).**

The figure legend has been changed to read: “*Subjects indicated whether it was titled clockwise (CW) or counter-clockwise (CCW) relative to vertical”*

*** Line 86 reads Shaded region indicates cortex not included in field of view." -- it's unclear to this region what shaded region refers to (on the flattened map?  On the hemisphere?)**

The figure legend now reads: “*Shaded region on lateral and medial views indicates cortex not included in the imaged field of view.”*

*** To make it clear to readers who may not be familiar with units of spatial frequency, the authors should rewrite cpd as cycles/deg in line 549.**

The text now reads: *“The stimulus consisted of a full contrast grating with a spatial frequency of 4 cycles/degree (cpd)”*

*** Citations are for the methods sections are sparse.  If based on techniques used in prior work, the authors should cite the relevant sources.**

We have added a number of citations in the Methods section.

*** Was there any eye tracking data showing that subjects were fixating centrally? If not, it's important to show data from the hemisphere where you expected activity to make sure that the activation in located in the location of the stimulus.**

Stimulus-evoked activity in the contralateral hemisphere was not robust (we designed the experiment to minimize stimulus-evoked responses). While we did perform eye tracking concurrent with fMRI scanning, it was of variable quality. We therefore analyzed the much higher quality eye tracking data from the pupil size experiment, which had sufficient spatial and temporal resolution to detect microsaccades and was stable over the course of the experiment. Subjects indeed fixated centrally, as instructed, and did not make saccades towards the stimulus (Results, first paragraph, and Fig S3).

***Fig S3. Saccades during pupil size experiment.*** *(A) Main sequence, pooling saccades across subjects, demonstrates a linear relationship in log-log axes between peak velocity and saccade amplitude, as expected from the biomechanics of the oculomotor plant. (B) Amplitude distribution of saccades. Most saccades were small (<1 deg), and are hence considered microsaccades. Median saccade amplitude, 0.52 deg. (C) Spatial distribution of saccades. Each dot represents the displacement of a saccade relative to the origin (0,0). (D) Direction distribution of saccades. Saccades were generally horizontal, and are were not directed toward the target, nor were they of sufficient amplitude to reach the target. Saccades occurring within the first 1000 ms of each trial were included in this analysis.*

**Reviewer #2: Task-related activity in human visual cortex**

**In this manuscript, the authors investigate the role that task based activity plays in modulating visuocortcial responses. To do so, they manipulate the value of accuracy-based reward on a fine orientation discrimination task, and find modulation of pupil diameter as a function of reward, consistent with an increase arousal.  Importantly, the authors also observed increases in BOLD response in early visual cortex, independent of regions of visual stimulation, and a decrease in trial-to-trial variability in BOLD response. The authors then propose a simple model wherein increased temporal precision can underlie their effects.  The topic is interesting and timely, and the study is generally well designed and analyses carefully conducted.  Below I outline my specific, albeit minor concerns.**

**1. Based on the pupil and heart rate measures, the authors conclude that their reward modulation was an effective driver of arousal.  While in principle I agree, the authors should note that they have not fully disentangled reward from arousal win this study. A pure effect of arousal would be evident in a valence-free modulation of pupil diameter and heart rate (and presumably BOLD response in visual cortex).  In other words, if the study was carried out with punishment rather than reward, the authors would expect the same modulatory effects, were they driven purely by arousal state.  Now, I don't think these observed effects in this study are *not* arousal-driven, but the authors should acknowledge that in this design reward has not been dissociated from arousal.**

We agree. We did not disentangle reward from arousal in this study and we have revised the text to reflect this. Our primary aim was to modulate arousal. In a sense, we linked arousal and reward by design. The physiological measures indicate that the reward manipulation worked. Pupil size and heart rate, both widely-used proxies for arousal, were elevated in high reward runs, indicating that arousal was higher as well. But, as the reviewer notes, it is likely the case that other experimental manipulations (e.g., cold-water shock or other punishment) would have similar effects on arousal levels. Punishment and reward may both increase arousal, and if so, they should both modulate task-related activity in a similar way, thereby disentangling reward and arousal. We now consider this issue in the Discussion (*Discussion: Manipulating arousal with reward*).

**2. There was a small, full contrast grating for which participants carried out an orientation discrimination task on.  And yet, the manuscript largely neglected to focus on any reward-related modulation of the stimulus-evoked BOLD response.  I get the impression there was no functional localizer carried out, but given that there was pRF mapping for the participants, would it be possible to look how the representation of the visual stimulus was affected by the reward value manipulation?**

This is an interesting question. This study was designed to minimize the impact of the visual stimulus. By using a small, brief stimulus, there was only very limited visually-driven activity detected even in the contralateral hemisphere in the region corresponding retinotopically to the location of the stimulus. All subjects were scanned in a functional localizer scan that identified the retinotopic location of the stimulus, and on separate trials, its mirror symmetric location in the opposite hemifield (see Fig S2). In the main experiment, the stimulus appeared in the lower quadrant of the right visual field. This location is identified in the localizer scan in Fig S2, left panel, blue response. There was only a very small response at this location in the main experiment (Fig S2, middle and right panels). Hence, it was not possible to characterize the impact of arousal on stimulus-driven activity.

In a separate study, we had begun pursuing this topic before the COVID-19 crisis, using a more robust, full-field stimulus that enabled us to characterize stimulus tuning properties (e.g., orientation, contrast, and spatial frequency). We are intrigued by a recent report that arousal modulates activity in mouse retina (Schroder et al., Neuron, 2020), suggesting that modulations with arousal are present in the very earliest stages of the visual system. We have added a discussion of these issues in the Discussion section of the revision (*Discussion: Effect of arousal on stimulus-evoked activity*) and we very much look forward to pursuing these and related questions once scanning of healthy human volunteers begins again at NIH.

**3. In line 55, authors state that "changes in arousal lead to pupil dilation and do not increase sensitivity [15, 20]."  This is not necessarily true. There are plenty of behavioral studies that have demonstrated improvements in perceptual performance with arousal state, including cognitive performance and low level perceptual sensitivity.**

The statement is based on a recent study that aimed to dissociate attention and arousal (Baruni et al., *Nature Neuroscience*, 2015). This study found that while attention increases sensitivity, arousal does not. Earlier studies that found an effect of arousal on sensitivity may have instead unintentionally measured an effect of attention (e.g., Phelps et al., *Psychological Science*, 2006; Kim et al., *Journal of Vision*, 2017). However, we agree that later studies may have missed effects of arousal on sensitivity, and this remains an important and interesting question. The text now reads: *“changes in arousal lead to pupil dilation and do not necessarily increase sensitivity [15, 20]”.*

**4.  Did the authors observe any correlation between the time series for pupil or heart rate, and BOLD modulation?  In other words, within a condition (High or low reward), were there fluctuations in physiological measures that squared with fluctuations in magnitude of BOLD response.**

The eye data measured concurrent with fMRI was of variable quality and did not enable us to test this correlation. Pulse did covary with the fMRI time series, and regressing pulse with BOLD yielded a clear and robust kernel. After performing global signal regression, this covariation was effectively eliminated (Fig 2). The correlation also dropped to near-zero after regression. The mean $r^{2}$ (at lag 0) after regression was 0.0000273.
