# Supplementary material for: Task-related activity in human visual cortex
Source: PLoS Biol. 2020 Nov 6;18(11):e3000921. doi: 10.1371/journal.pbio.3000921 (PMC7673548; doi:10.1371/journal.pbio.3000921)
Supplement: Attachment — Submitted filename: PLOS_response.docx [file pbio.3000921.s008.docx]

We would appreciate your prompt cooperation with our requests at your earliest convenience. If you have any questions or require clarification, please do not hesitate to get in touch.

1.	Please indicate the corresponding author on the title page with an asterisk (*) and include the corresponding author's email address. Only an asterisk and the email address itself are required.

On the title page, corresponding author is indicated by asterisk, and corresponding author’s email is included.

2.	We have noted that you have the following present in your manuscript: Data Availability Statement (“Data and code sharing”)

We have removed these section from your manuscript file as this information is automatically pulled from the submission form. Please make sure that the information in the submission form is correct.

This section has been removed, and the information has been confirmed.

3.	Thank you for uploading your data to OSF. Please also include a README file that explains how these data were analyzed to generate the plots and graphs you show in Figures 2A-C, 3, 4A-C, 5A-C, 6A-H, 7AB, S1B, S2BD, S4A-C, and S5A-C.

A README file is now included as an OSF Wiki.

4.	Please also ensure that each figure legend in your manuscript include information on where the underlying data can be found (<https://doi.org/10.17605/osf.io/cbjq6>)

All figure legends (Except for Fig 6, with no data) now include information on where the underlying data is found.

5.	Please confirm that you either created the following figure panels, or that you will replace them with an open source alternative: Fig 1, panels A and B, S1 Fig, panel A

Here are a few open source image websites you may wish to use:

- <https://commons.wikimedia.org>

- <http://www.publicdomainpictures.net/>

- <https://openclipart.org/>

Before resubmitting, please check to make sure you have added all submission files to the File Inventory, as missing files will result in further delays.

I confirm that I created all figures, including Fig 1, panels A and B, S1 Fig, panel A
